# Supplementary material for: An open-source framework for large-scale, flexible evaluation of biomedical text mining systems
Source: J Biomed Discov Collab. 2008 Jan 29;3:1. doi: 10.1186/1747-5333-3-1 (PMC2276192; doi:10.1186/1747-5333-3-1)
Supplement: Additional file 1 — Evaluation platform source code. The additional file contains the software evaluation framework discussed in this paper. The most current release can be downloaded from SourceForge.net [1]. See the accompanying README file for instructions on installation and use. Also included in the distribution is a collection of UIMA wrappers for some commonly used BioNLP tools and annotated corpora. [file 1747-5333-3-1-S1.zip › bionlp_uima_repository-1.0.1/docs/api/src-html/edu/uchsc/ccp/uima/ae/util/filter/AnnotationConsensusFilter_AE.html]

```
001    /*
002     * Created on Sep 8, 2005
003     *
004     * TODO To change the template for this generated file go to
005     * Window - Preferences - Java - Code Style - Code Templates
006     */
007    package edu.uchsc.ccp.uima.ae.util.filter;
008    
009    import java.util.Arrays;
010    import java.util.HashMap;
011    import java.util.HashSet;
012    import java.util.Iterator;
013    import java.util.Map;
014    import java.util.Set;
015    
016    import org.apache.uima.analysis_engine.ResultSpecification;
017    import org.apache.uima.analysis_engine.annotator.AnnotatorConfigurationException;
018    import org.apache.uima.analysis_engine.annotator.AnnotatorContext;
019    import org.apache.uima.analysis_engine.annotator.AnnotatorContextException;
020    import org.apache.uima.analysis_engine.annotator.AnnotatorInitializationException;
021    import org.apache.uima.analysis_engine.annotator.AnnotatorProcessException;
022    import org.apache.uima.analysis_engine.annotator.JTextAnnotator_ImplBase;
023    import org.apache.uima.cas.CASException;
024    import org.apache.uima.jcas.JCas;
025    import org.apache.uima.jcas.cas.FSArray;
026    
027    import edu.uchsc.ccp.uima.annotation.CCPAnnotationSet;
028    import edu.uchsc.ccp.uima.annotation.CCPAnnotator;
029    import edu.uchsc.ccp.uima.annotation.CCPTextAnnotation;
030    import edu.uchsc.ccp.uima.mention.CCPClassMention;
031    import edu.uchsc.ccp.uima.util.UIMA_Util;
032    
033    /**
034     * @author williamb Takes longer time to run the first time.. not sure why
035     */
036    public class AnnotationConsensusFilter_AE extends JTextAnnotator_ImplBase {
037        private final boolean DEBUG = false;
038    
039        private int consensusThreshold = 1;
040    
041        public final String PARAM_ANNOTATION_SETS_TO_IGNORE = "AnnotationSetsToIgnore";
042    
043        /**
044         * The annotation type to look at when making consensus selections
045         */
046        public final String PARAM_ANNOTATION_TYPE_OF_INTEREST = "AnnotationTypeOfInterest";
047    
048        /**
049         */
050        public static final String PARAM_CONSENSUS_THRESHOLD = "ConsensusThreshold";
051    
052        protected Set<Integer> annotationSetsToIgnore;
053    
054        protected String annotationTypeOfInterest;
055    
056        public void initialize(AnnotatorContext ac) throws AnnotatorInitializationException, AnnotatorConfigurationException {
057    
058            try {
059                consensusThreshold = ((Integer) ac.getConfigParameterValue(PARAM_CONSENSUS_THRESHOLD)).intValue();
060                annotationTypeOfInterest = (String) ac.getConfigParameterValue(PARAM_ANNOTATION_TYPE_OF_INTEREST);
061                try {
062                annotationSetsToIgnore = new HashSet<Integer>(Arrays.asList((Integer[]) ac
063                        .getConfigParameterValue(PARAM_ANNOTATION_SETS_TO_IGNORE)));
064                } catch (NullPointerException npe) {
065                    annotationSetsToIgnore = new HashSet<Integer>();
066                }
067            } catch (AnnotatorContextException ace) {
068                ace.printStackTrace();
069            }
070            
071            System.err.println("Initialized Consensus Filter with threshold=" + consensusThreshold);
072            
073            super.initialize(ac);
074        }
075    
076        public void process(JCas jcas, ResultSpecification arg1) throws AnnotatorProcessException {
077            /* cycle through all protein annotations, and look for ones that satisfy the consensus threshold for agreement */
078            Map<String, Integer> span2countMap = new HashMap<String, Integer>();
079    
080            Iterator annotIter = jcas.getJFSIndexRepository().getAnnotationIndex(CCPTextAnnotation.type).iterator();
081            while (annotIter.hasNext()) {
082    
083                CCPTextAnnotation ccpTA = (CCPTextAnnotation) annotIter.next();
084                boolean ignore = checkForIgnoreBasedOnAnnotationSet(ccpTA);
085    
086                if (!ignore) {
087                    if (ccpTA.getClassMention().getMentionName().equals(annotationTypeOfInterest)) {
088    
089                        String spanStr = ccpTA.getBegin() + "|" + ccpTA.getEnd();
090                        if (span2countMap.containsKey(spanStr)) {
091                            int count = span2countMap.get(spanStr);
092                            count++;
093                            span2countMap.remove(spanStr);
094                            span2countMap.put(spanStr, new Integer(count));
095                        } else {
096                            span2countMap.put(spanStr, new Integer(1));
097                        }
098                    }
099                }
100            }
101    
102            /* cycle through the span2countmap and output annotations that pass the consensus threshold */
103            Set<String> spanStrings = span2countMap.keySet();
104            for (String spanStr : spanStrings) {
105                int countForThisSpan = span2countMap.get(spanStr);
106                if (countForThisSpan >= consensusThreshold) {
107                    /* then create a new annotation */
108                    String[] toks = spanStr.split("\\|");
109                    createConsensusAnnotation(Integer.parseInt(toks[0]), Integer.parseInt(toks[1]), jcas);
110                }
111            }
112    
113        }
114    
115        private void createConsensusAnnotation(int startIndex, int endIndex, JCas jcas) {
116            CCPTextAnnotation consensusAnnotation = new CCPTextAnnotation(jcas);
117            try {
118                UIMA_Util.setCCPTextAnnotationSpan(consensusAnnotation, startIndex, endIndex);
119            } catch (CASException e1) {
120                e1.printStackTrace();
121            }
122    
123            /* set annotator and annotation set */
124            CCPAnnotator ccpAnnotator = new CCPAnnotator(jcas);
125            ccpAnnotator.setAffiliation("UCHSC");
126            ccpAnnotator.setFirstName("Consensus Filter");
127            ccpAnnotator.setAnnotatorID(88);
128            ccpAnnotator.setLastName("Consensus Filter");
129    
130            CCPAnnotationSet ccpAnnotationSet = new CCPAnnotationSet(jcas);
131            ccpAnnotationSet.setAnnotationSetID(88);
132            ccpAnnotationSet.setAnnotationSetName("Consensus Annotations");
133            ccpAnnotationSet.setAnnotationSetDescription("");
134    
135            consensusAnnotation.setAnnotator(ccpAnnotator);
136            FSArray asets = new FSArray(jcas, 1);
137            asets.set(0, ccpAnnotationSet);
138            consensusAnnotation.setAnnotationSets(asets);
139    
140            consensusAnnotation.setDocumentSectionID(-1);
141    
142            CCPClassMention ccpCM = new CCPClassMention(jcas);
143            ccpCM.setMentionName(annotationTypeOfInterest);
144            consensusAnnotation.setClassMention(ccpCM);
145            try {
146                UIMA_Util.addCCPTextAnnotationToCCPClassMention(consensusAnnotation, ccpCM);
147            } catch (CASException e) {
148                e.printStackTrace();
149            }
150            consensusAnnotation.addToIndexes();
151    
152          
153    
154        }
155    
156        protected boolean checkForIgnoreBasedOnAnnotationSet(CCPTextAnnotation ccpTA) {
157            boolean ignore = false;
158            FSArray annotationSets = ccpTA.getAnnotationSets();
159            for (int i = 0; i < annotationSets.size(); i++) {
160                CCPAnnotationSet aSet = (CCPAnnotationSet) annotationSets.get(i);
161                if (annotationSetsToIgnore.contains(aSet.getAnnotationSetID())) {
162                    ignore = true;
163                    break;
164                }
165            }
166            return ignore;
167        }
168    
169    }
```
